# Garden Access, Race and Vegetable Acquisition among U.S. Adults: Findings from a National Survey

**DOI:** 10.3390/ijerph182212059

**Published:** 2021-11-17

**Authors:** Joelle N. Robinson-Oghogho, Roland J. Thorpe

**Affiliations:** Department of Health Behavior and Society, Bloomberg School of Public Health, Johns Hopkins University, Baltimore, MD 21205, USA; rthorpe@jhu.edu

**Keywords:** disparities, food access, vegetable intake

## Abstract

With the majority of U.S. adults not meeting recommended vegetable intakes and well-documented racial and ethnic disparities in fruit and vegetable consumption, various approaches to increase vegetable consumption have been implemented. Gardening is one approach that has been associated with increased vegetable consumption in various subpopulations; however, limited national data exist examining this relationship. Since vegetable acquisition is a necessary antecedent to increased vegetable consumption, this study examines if garden access is associated with vegetable acquisition among adults in a nationally representative sample of U.S. households. Data come from the National Food Acquisition and Purchasing Survey (FAPS), a survey of 4826 US households. Descriptive analysis and modified Poisson regressions were performed to examine associations between household garden access and vegetable acquisition amongst the total population and by race. Results indicate that for foods for at-home consumption, respondents with their own garden had a 30% greater prevalence (PR: 1.30, 95% CI: 1.01, 1.64) of acquiring enough vegetables to meet USDA recommendations compared to respondents in households without access to any gardens. Among Black respondents, those with access to their own garden had over two times increased prevalence (PR: 2.35, 95% CI: 1.10, 5.01) of acquiring enough vegetables to meet recommended consumption amounts, compared to Black respondents without any access to a garden. No relationships between garden access and vegetable acquisition were observed for White or Asian respondents. This information may contribute to the body of evidence on strategies for increasing vegetable consumption among U.S. adults.

## 1. Introduction

It has been estimated that only 9% to 13% of U.S. adults meet the recommended vegetable intakes [1,2]. Meanwhile, racial and ethnic disparities in overall health and fruit and vegetable consumption have been well documented [3,4,5]. In the U.S., people identifying as Black have lower rates of vegetable consumption and poorer nutritional quality compared to their White counterparts [6,7]. A number of factors have been studied as contributing to variations in vegetable consumption, such as individual perceptions of the taste, preparation time, cost, and accessibility of fruits and vegetables [8,9]. Additionally, socioeconomic, demographic, and community-level variables factors such as race, gender, income, education, and proximity to grocery stores or healthy food markets often intersect to enforce diet-related disparities [10,11].

For example, racial segregation has been analyzed as the social stratification process that shapes food density in urban areas [12]. It has been well documented that the foods available in low-income areas or neighborhoods with higher proportions of Black residents are often less healthy than foods available in Whiter or higher-income areas [13,14]. Systemic barriers to healthy food access, such as lack of neighborhood supermarkets disproportionately impact neighborhoods with higher proportions of people of color and people with lower incomes [5,15]. 

Improving an individual’s ability to acquire vegetables is a necessary antecedent to increased vegetable consumption. As most U.S. consumers acquire their foods from supermarkets [16], the distribution of food retail across places becomes an important, albeit not the only factor, influencing what kinds of foods people can obtain. Lower numbers of supermarkets and high density of fast-food restaurants have been frequently identified as features of the built and social environment negatively associated with vegetable consumption [5,13,17]. The resulting socially constructed conditions of food apartheid, founded in race-based community segregation policies such as redlining [18,19], continue to negatively impact many predominantly black and brown communities across the country. 

A variety of initiatives aimed at increasing vegetable consumption have been implemented across the U.S., including multi-pronged initiatives aimed at changing the food environment to increase access to fruits and vegetables, both in terms of affordability and geographic proximity [20]. Some interventions to increase vegetable consumption emphasize connecting consumers to agricultural food production, such as direct farmer sales via farmer markets or Community Support Agriculture shares (CSAs), and/or supporting home and community gardening [21,22]. Although supermarkets are a major source of food acquisition [16], people also obtain food outside traditional retail outlets. Studies suggest that consumer participation in the food system (i.e., production, processing, distribution) is associated with increased vegetable consumption [23], and gardening specifically has been identified as a facilitator for increasing vegetable consumption among U.S. children [24,25] and adults [26,27]. Growing food directly has the potential to supplement household food costs while also signifying other social, emotional, or cultural importance [28,29]. 

Despite the potential benefits of gardening, there may be differential participation or access to opportunities to garden among different populations. For example, the systematic limiting of access to land ownership and tenure based on race has been well documented in the U.S. [30]. It is possible that these impediments to land access have led to race differences in gardening or access to gardens for food production. Few studies have focused on the relationship between household access to home or community gardens and household vegetable availability by race at a population level. As household food environment is a key factor influencing vegetable consumption [31,32], identifying how alternative food acquisition strategies that support vegetable consumption differ among subpopulations may provide insight into potential intervention opportunities. 

Additionally, very few studies exist in the peer-reviewed literature that rigorously examines the impact of gardening activities on vegetable consumption among U.S. adults [33,34]. Much of the research on the relationship between gardening and vegetable consumption has focused on specific communities [34,35,36,37,38] or child populations [39,40]. Although developing food preference at a young age is important, adult household members are usually responsible for food purchasing and determining the types of food made available in the household. 

National data has not been explored to examine the relationship between access to gardens for food production and vegetable acquisition or consumption. The USDA National Food Acquisition and Purchasing Survey (FoodAPS) collects information on food acquisition from sources not measured in other U.S. food purchasing surveys, such as gardening and hunting. Using data from FoodAPS, this analysis aims to explore if garden access is associated with vegetable acquisition among adults in a nationally representative sample of U.S. households and examine these relationships by race. This information may contribute to the body of evidence on strategies for increasing vegetable consumption among U.S. adults.

## 2. Materials and Methods

### 2.1. Data Source

Data are from the National Household Food Acquisition and Purchasing Survey (FoodAPS), a nationally representative survey of 4826 US households. Data collection occurred from April 2012 through mid-January 2013. The FAPS was sponsored by the U.S. Department of Agriculture (USDA) and managed by USDA’s Economic Research Service (ERS). Additional information on FAPS can be found at https://www.ers.usda.gov/data-products/foodaps-national-household-food-acquisition-and-purchase-survey/documentation/ (accessed on 30 March 2019).

The household’s main food shopper or meal planner was selected to be the primary survey respondents and was asked questions about the household and its members. Each household member, age 11 years and older, was asked to track and provide information on the foods they acquired over a 7-day period. Participants used booklets to record their food acquisitions during the 1-week data collection period. This analysis examines data from the 4826 primary respondents and does not include data from other household members. 

FoodAPS includes data on foods acquired at home (FAH) and those acquired away from home (FAFH). The food at home category generally includes items obtained from grocery stores, supermarkets, supercenters, gardens, or farmers’ markets, whereas foods away from home are generally purchased at restaurants or other prepared food vendors [41]. The distinction between food at home and food away from home is made because foods away from home are known to be less nutritious or healthy than foods prepared at home [42,43]. With this in mind, three separate vegetable acquisition variables were created for foods obtained for home consumption, foods obtained for consumption away from home, and all foods combined (consisting of foods at home and away from home). 

### 2.2. Measures

Vegetable acquisition is the main outcome of interest. Total vegetable acquisition among the primary respondent for each household is defined as the total cup equivalent per 100 g of dark green, red and orange, starchy, and other vegetables, excluding legumes. A participant’s total vegetable acquisition was generated by calculating the sum of all vegetable components for each food item recorded during the data collection period. Each primary respondent’s total vegetables was then divided by the sum of their total energy calories acquired during the 7-day data collection period and multiplied by 2000. This gives the total cups of vegetables per 2000 calories. This method is similar to what has been used in previous studies [42,44] and aligns with the USDA dietary guidelines for adults [45]. A binary variable was then constructed to identify respondents who acquired enough vegetables to meet the USDA recommended vegetable consumption for adults. Participants acquiring 0 to 2.49 cups of vegetables per 2000 calories were defined as not acquiring enough vegetables to meet recommended vegetable consumption. Participants acquiring 2.5 or more cups of vegetables per 2000 calories are defined as meeting or exceeding recommendations.

Access to gardens, the main independent variable, was captured at the household level in two dichotomous variables. Respondents were asked if their household “has a vegetable garden when in season” and if they “receive fruits or vegetables from anyone else’s garden”. For this analysis, these two variables were combined into one categorical variable with the following four groups: “No Access,” “Access to Someone Else’s Only,” “Access to Own Only,” and “Access to Own and Someone Else’s.” Here, household access was used as a proxy for gardening. It is presumed that people in households reporting access to their own gardens or receiving fruits and vegetables from someone else’s garden have at least one household member that furnishes the household with produce from their gardening activities or engagements. 

To assess race, primary respondents were asked to select all the race categories that they identified as. Racial categories included “White”, “Black or African American”, “American Indian or Alaska Native”, “Asian, Native Hawaiian (NH) or Other Pacific Islander (PI)”, and “Other”. For this analysis, race was based on self-report and categorized as White, Black, and Asian, Native Hawaiian, or Pacific Islander. Participants who identify as American Indian/Alaskan Native (n = 43) and Other or multiple races (n = 490) were excluded from these analyses. Participants in each of these categories account for 0.44% and 6.5% of the weighted percentage of the total population of analysis, respectively. 

Additional demographic and behavioral variables which have been known to be associated with vegetable consumption were also included in these analyses. Demographic variables include socioeconomic status, vegetarian diet, age, sex (e.g., male, female), rural geography (e.g., rural, non-rural), and household size. At the same time, behavioral variables refer to respondents’ perceptions of healthy eating. To assess perceptions of healthy eating, the primary respondent were asked to agree or disagree with the following statements, “It costs too much for (me/my family) to eat healthy foods,” “I’m too busy to take the time to prepare healthy foods,” and “I don’t think healthy foods taste good.” Socioeconomic status variables include educational attainment (e.g., less than high school, high school graduate or general educational diploma (GED), some college, college graduate or professional degree), and household income. Monthly household income was categorized into quintiles of the income range of the total study population (e.g., $0–$1119, $1120–$2099, $2100–$3499, $3500–$5628, $5629–$25,000). A vegetarian diet was indicated as “yes” or “no”. Age was categorized into three groups, 16–35 years, 36–59 years, and 60 years and older. Only three primary respondents were below the age of 18. Household size was categorized into three groups based on the total number of household members (e.g., 1–2 persons, 3–5 persons, 6 or more persons). 

### 2.3. Analytic Approach

Descriptive analyses were conducted to examine the vegetable acquisition for food at home and food away from home purchases by household garden access status among the total population. Twelve percent of primary respondents did not have information on the amount of vegetables acquired for foods to be consumed at home, and 18% of primary respondents had missing data for vegetable acquisition for foods for consumption away from home. Respondents with missing data on vegetable acquisition were excluded from the analysis. Descriptive analysis and chi-square test were conducted to determine if there were significant differences in the proportional distribution garden access by race and all other covariates. Missing data for covariates accounted for less than 1% and were not included in the descriptive analysis. 

Modified Poisson regressions were then used to estimate the prevalence rate ratios for acquiring enough vegetables to meet USDA recommendations for adult vegetable consumption by household garden access while adjusting for demographic and behavioral covariates. Modified Poisson regressions have been used to provide estimates of prevalence ratios for binary outcomes in cross-sectional studies when the outcome is not rare [46,47,48], as was the case in our analysis. These regression analyses were conducted among the total population and then stratified by race, given what is currently known about historical and contextual factors impacting opportunities for vegetable acquisition by race. 

All analyses were weighted to account for complex sampling design and support estimations for the contiguous United States. The jackknife repeated replication technique was used to produce weighted analysis applying the replicate weights provided in the data files and outlined in the survey documentation [49]. All analyses were conducted using STATA 15.1 (StataCorp LLC, College Station, TX, USA) [50]. *p*-values less than 0.05 were considered statistically significant. 

## 3. Results

The distribution of household garden access by select demographic and behavioral characteristics is shown in Table 1. Among all respondents, 51% live in households with any type of access to a garden, including access to someone else’s garden only, access to their own garden, or both access to their own and someone else’s garden. Differences in garden access vary across race groups, with 57% of White respondents living in a household with access to a garden and only 32% Black and Asian respondents living in households with access to gardens. A greater proportion of women (55.3%) and people living in rural areas (69.4%) reported having access to their own or someone else’s garden.

The large majority of respondents (over 74%) did not acquire enough vegetables during the week to meet the recommended intake of 2.5 cups of vegetables per 2000 calories. As seen in Table 1, the bivariate analysis did not show any differences in household garden access among respondents acquiring enough vegetables to meet USDA consumption recommendations for foods purchased for at-home consumption, away from home consumption, and both at home and away from home consumption. 

The association between vegetable acquisition and garden access in the total population is shown in Table 2. After controlling for race, sex, geography, household income, educational attainment, age group, taste, time and cost perceptions of healthy foods, vegetarian diet, and household size, respondents with their own garden had a 30% greater prevalence of acquiring enough vegetables to meet USDA recommendations when examining foods for at-home consumption (PR: 1.30, 95% CI: 1.01, 1.64) compared to people in households without access to any gardens. 

When examining the relationship between garden access and vegetable acquisition stratified by race, results indicate that household garden access may positively influence vegetable acquisition among Black respondents. After controlling for sex, geography, household income, educational attainment, age group, taste perceptions, time perceptions, cost perceptions, and vegetarian diet, Black respondents with access to their own garden had over two times increased prevalence of acquiring at least 2.5 cups of vegetables in the foods for at-home consumption (PR: 2.35, 95% CI: 1.10, 5.01) than other Black people without any access to a garden. No relationships between garden access and vegetable acquisition were observed for White or Asian respondents. 

## 4. Discussion

The objective of this analysis was to use data from a nationally representative sample of U.S. households to examine if having access to a garden is associated with vegetable acquisition and examine this relationship by race. The findings on the vegetable acquisition were consistent with the existing literature that most U.S. adults do not consume the recommended amount of vegetables [1,2]. 

This analysis provides evidence to suggest that household access to gardens may affect the vegetable acquisition behaviors of household members who are the main food shoppers or meal planners. Adults who lived in households with access to their own gardens had nearly 30% higher rates of acquiring enough vegetables for foods they consumed at home to meet USDA consumption recommendations than adults who reported not having access to a garden. Having a home garden may be a useful tool for improving household availability of vegetables. As the relationship between garden access and vegetable acquisition was only seen for foods acquired at home, people who have greater access to gardening and vegetables at home may be more likely to choose food items that do not contain vegetables when they are food shopping or eating on the run (e.g., acquire foods away from home). 

Our findings also showed that Black respondents who had access to their own garden had rates two times higher than Black respondents without access to a garden for acquiring enough vegetables to meet USDA consumption recommendations. For over two decades, food justice researchers and advocates have identified systematic barriers to accessing healthy food options for many low income and black and brown populations, such as lack of grocery or healthy food retail locations in consumers neighborhood food environments as well as limited access to land [5,12,13,14,15,51]. The results of this study suggest that home gardens may be a useful tool for improving vegetable acquisition, particularly among respondents who identify as Black. 

This study has several limitations. The data on which this analysis is based was collected several years prior to this analysis and publication. Since then, the global COVID-19 pandemic has impacted aspects of food acquisition and created economic challenges. There is some evidence that this has resulted in increased gardening and home food production [52]. More recent data collection on the measures analyzed here would further understand the relationship between gardening and vegetable acquisition in the current context. Additionally, as food access is often considered in terms of food affordability and proximity to retail locations, accounting for differences in the geo-special availability of opportunities to purchase health foods could have been an important covariate to include. Although the FoodAPS data does include information on distance and travel time to a household’s primary food shopping location, we recognize the challenges and complexities of using distance variables to measure “access” to healthy food [53] and decided not to include them. It should also be noted that this analysis is based on food procurement behaviors and not consumption. Given the robust body of research disparities related to vegetable consumption rather than procurement, the covariates included in this analysis were based on the current research around factors influencing vegetable consumption. Nonetheless, food acquisition or procurement remains a necessary antecedent to consumption, so it is reasonable that factors influencing consumption may also influence acquisition behaviors. A final limitation of this analysis is that it did not fully account for household composition. Although this analysis does include information on household size, it is likely that individuals have different food acquisition patterns depending on the number and ages of members in their household. 

Despite these limitations, this study provides insights into how access to food acquisitions outside of traditional retail may influence vegetable procurement patterns in a national sample. The information gained from this analysis provides some evidence that gardening likely improves the household food environment. Additionally, the stratification of this analysis by self-reported race categories is another strength. By analyzing the relationship between garden access and vegetable acquisition within race groups rather than using one racial group as the reference to compare other groups, this approach recognizes that racial identities are often deeply connected with socioeconomic factors and historical and ongoing processes of social stratification, thereby resulting in differential life experiences by racial identity. This analysis addresses the paucity of research examining the impact of gardening activities on vegetable consumption among U.S. adults and takes that analysis a step further by examining this relationship within race categories. 

## 5. Conclusions

In conclusion, it is conceivable that gardening and household access to gardens address several domains of food access, including economic cost, time and space, and fostering social networks and cultural foodways. When considering the effect of nutrition interventions that have gardening components compared to interventions without [54,55], gardening may provide an experiential connection to fruit and vegetable production in ways that may not be present in other behavioral or environmental interventions seeking to improve vegetable consumption. The findings of this study suggest that creating opportunities and encouraging households to garden may continue to be a helpful intervention for increasing household availability and subsequent vegetable consumption. Careful intention and consideration should be made to ensure that such interventions are historically grounded and culturally relevant. 

## Figures and Tables

**Table 1:** Weighted distribution of household garden access by select behavioral and demographic characteristics in the National Household Food Acquisition and Purchase Survey (FoodAPS).

	%	Garden Access %				
Total Pop. N = 4826		No Access N = 2715	Someone Else’s Only N = 962	Own Only N = 594	Own & Someone Else N = 555	*p*-Value
**Food at Home** n = 4229						0.189
Veg. Acquisition ≥ Recommendations	26.4	44.0	21.4	17.3	17.3	
**Food Away from Home** n = 3949						0.804
Veg. Acquisition ≥ Recommendations	24.5	49.7	21.1	14.5	14.7	
**Food at Home & Away from Home** n = 4650						0.221
Veg. Acquisition ≥ Recommendations	24.4	49.1	19.4	16.3	15.3	
**Race** n = 4287						**<0.001**
White	81.9	43.0	23.1	17.1	16.9	
Black	13.6	68.5	20.7	5.0	5.8	
Asian, NH, PI	4.5	68.0	14.6	8.3	9.1	
**Sex** n = 4826						**0.003**
Women	67.6	44.7	23.9	15.4	16.0	
Men	32.4	57.8	18.0	12.9	11.3	
**Geography** n = 4826						**<0.001**
Rural	33.8	30.6	28.3	21.2	20.0	
Non-Rural	66.2	58.3	18.8	11.2	11.7	
**Household Monthly Income** n = 4801						**<0.001**
$0–$1119	18.6	62.1	18.1	9.6	10.2	
$1120–$2099	14.3	53.6	27.5	11.0	7.9	
$2100–$3499	16.1	46.9	22.4	17.6	13.1	
$3500–$5628	22.8	42.8	21.9	16.3	19.1	
$5629–$25,000	28.3	43.8	22.2	16.8	17.2	
**Educational Attainment** n = 4823						<0.057
Less than High School	9.7	62.5	13.2	14.4	10.0	
High School or GED	24.7	47.7	22.5	15.2	14.7	
Some College	33.3	46.7	23.3	14.0	16.0	
College Degree/Professional Degree	32.4	48.2	22.9	14.8	14.1	
**Age Category** n = 4826						**<0.001**
16–35	23.8	58.1	23.9	8.4	9.6	
36–59	46.0	47.3	22.0	14.5	16.2	
60+	30.3	44.3	20.5	19.4	15.8	
**Healthy Food Taste Bad** n = 4823						**0.017**
Agree	11.1	60.3	19.9	11.8	8.0	
**Healthy Food Too Much Time** n = 4823						**<0.001**
Agree	21.7	53.5	26.1	10.5	9.9	
**Healthy Food Cost Too Much** n = 4825						**0.003**
Agree	33.0	53.7	22.9	12.9	10.5	
**Vegetarian** n = 4826						0.677
Yes	3.0	55.3	19.6	13.6	11.5	
**Household Size** n = 4822						0.189
1–2 persons	61.4	49.1	22.3	14.9	13.7	
3–5 persons	34.7	48.1	22.2	13.4	16.3	
6 or more persons	3.8	54.3	16.0	20.2	9.6	

Bolded values indicate statistically significant difference at a 95% confidence interval.

**Table 2:** Adjusted associations between garden access and primary respondent vegetable acquisitions in the National Household Food Acquisition and Purchase Survey (FoodAPS) for total population and stratified by race.

	Prevalence Rate Ratio of Acquiring Vegetables to Meet USDA Recommended Intake (≥2.5 Cup Equivalents Per 2000 Calories)		
	Food at Home	Foods Away from Home	Total Food
	Prevalence Ratio (95% CI)	Prevalence Ratio (95% CI)	Prevalence Ratio 95% CI
Total Population *			
**Garden Access**	N = 3739	N = 3131	N = 3739
No Access (Ref)			
Access to Someone Else’s Only	1.07 (0.82, 1.39)	0.87 (0.63, 1.20)	0.93 (0.072, 1.19)
Access to Own Only	**1.28** **(1.01, 1.63)**	0.91 (0.64, 1.30)	1.20 (0.89, 1.60)
Access to Own and Someone Else’s	1.22 (0.96, 1.54)	0.84 (0.64, 1.11)	1.09 (0.91, 1.30)
**Race: White**			
**Garden Access**	N = 3006	N = 3722	N = 3006
No Access (Ref)			
Access to Someone Else’s Only	0.97 (0.74, 1.28)	0.94 (0.69, 1.30)	0.84 (0.64, 1.08)
Access to Own Only	1.21 (0.92, 1.58)	0.96 (0.66, 1.38)	1.14 (0.83, 1.56)
Access to Own and Someone Else’s	1.14 (0.86, 1.52)	0.90 (0.66, 1.21)	1.07 (0.89, 1.28)
**Race: Black**			
**Garden Access**	N = 547	N = 462	N = 547
No Access (Ref)			
Access to Someone Else’s Only	1.43 (0.67, 3.07)	0.73 (0.37, 1.42)	1.16 (0.60, 2.26)
Access to Own Only	**2.35** **(1.10, 5.01)**	0.68 (0.32, 1.45)	1.60 (0.75, 3.46)
Access to Own and Someone Else’s	2.16 (0.81, 5.84)	0.44 (0.11, 1.79)	0.83 (0.25, 2.76)
**Race: Asian/NA/PI**	N = 186	N = --	N = 186
**Garden Access**			
No Access (Ref)			
Access to Someone Else’s Only	1.22 (0.74, 2.04)	--	1.14 (0.602, 2.16)
Access to Own Only	1.37 (0.68, 2.77)	--	1.50 (0.96, 2.35)
Access to Own and Someone Else’s	0.81 (0.33, 1.96)	--	0.88 (0.41, 1.94)

Bolded values indicate statistically significant difference at a 95% confidence interval. All analysis controlled for sex, rural geography, household income, educational attainment, age group, taste perceptions, time perceptions, cost perceptions, vegetarian diet, and household size. * Total population model controls for respondent race in addition to the covariates mentioned above. -- Sample for subpopulation too small to produce estimates using the described Poisson model. The model did not converge.

## Data Availability

Publicly available data were analyzed in this study. Data supporting the results reported in this manuscript are available in a publicly accessible repository. The data can be found here: https://www.ers.usda.gov/data-products/foodaps-national-household-food-acquisition-and-purchase-survey/ (accessed on 30 March 2019). [dataset] USDA Economic Research Service. (2016). FoodAPS National Household Food Acquisition and Purchase Survey. Economic Research Service, Department of Agriculture. https://data.nal.usda.gov/dataset/foodaps-national-household-food-acquisition-and-purchase-survey (accessed on 30 March 2019).
